# Long-term plasticity of NMDA GluN2B (NR2B) receptor in anterior cingulate cortical synapses

**DOI:** 10.1177/17448069241230258

**Published:** 2024-02-07

**Authors:** Min Zhuo

**Affiliations:** 1 School of Basic Medical Sciences, Fujian Medical University, Fuzhou, China; 2 Qingdao International Academician Park, Zhuomin Institute of Brain Research, Qingdao, China; 3 Department of Physiology, 7938 University of Toronto, Toronto, ON, Canada

**Keywords:** Anterior cingulate cortex, long-term depression, N-methyl-D-aspartate receptor, post-long-term potentiation, pre-long-term potentiation

## Abstract

The anterior cingulate cortex (ACC) is a key cortical area for pain perception, emotional fear and anxiety. Cortical excitation is thought to be the major mechanism for chronic pain and its related emotional disorders such as anxiety and depression. GluN2B (or called NR2B) containing NMDA receptors play critical roles for such excitation. Not only does the activation of GluN2B contributes to the induction of the postsynaptic form of LTP (post-LTP), long-term upregulation of GluN2B subunits through tyrosine phosphorylation were also detected after peripheral injury. In addition, it has been reported that presynaptic NMDA receptors may contribute to the modulation of the release of glutamate from presynaptic terminals in the ACC. It is believed that inhibiting subtypes of NMDA receptors and/or downstream signaling proteins may serve as a novel therapeutic mechanism for future treatment of chronic pain, anxiety, and depression.

## Introduction

Adult mammalian brains are highly plastic. There are two major forms of synaptic plasticity in most of central synapses: long-term potentiation (LTP) and long-term depression (LTD). While most of excitatory synaptic responses are mediated by postsynaptic AMPA receptors, the N-methyl-D-aspartate (NMDA) receptor is a major type of ionotropic glutamate receptor for the induction of synaptic plasticity. Changes in synaptic responses during LTP/LTD are mainly carried out by AMPA receptors, as well as changes in the release of glutamate from presynaptic terminals. This review will focus on NMDA receptors in the anterior cingulate cortex (ACC). ACC is believed to contribute to the perception of pain and emotional responses. Glutamate is the major neurotransmitter in the ACC.^1,2^ In particular, this review will focus on the progress made in our understanding of NMDA receptors containing GluN2B (or NR2B). The contribution of this subtype to synaptic transmission and plasticity in the ACC will be discussed, as well as peripheral injury triggered changes of AMPA receptors in cortical synapses, and long-term upregulation of NMDA GluN2B receptors. Possible clinical significance for using GluN2B as a treatment target for chronic pain will be discussed.

## NMDA receptor and GluN2B

The NMDA receptor is a major type of ionotropic glutamate receptor in the brain, and can contribute to both forms of synaptic plasticity.^3–6^ NMDA receptors are more permeable to Ca^2+^ as compared with other glutamate receptors, and it is well known that Ca^2+^ serves as an important intracellular signaling molecule for inducing postsynaptic and presynaptic long-term changes. There are three family members: GluN1, GluN2 (GluN2A, -2B, -2C and -2D), and GluN3 (GluN3A, GluN3B). In central synapses, glutamate NMDA receptors are assembled in tetrameric form. Two GluN1 subunits and two GluN2 and/or GluN3 subunits combine together to form di-heteromeric or tri-heteromeric receptors. The composition of the subunits may change during neurodevelopment.^6,7^ NMDA receptors mediated currents show slower kinetics which allows longer permeability of Ca^2+^. By combining different pharmacological and genetic approaches, various NMDA receptor subtypes have been identified, and their roles in synaptic plasticity have been investigated. In adult central synapses, NMDA receptors contain heteromeric combinations of two GluN1 subunits, plus two of the GluN2A-D subunits.^7^

The properties of central NMDA receptors – including the sensitivity to protons, polyamines, and Zn^2+^- are determined by the splice variant of GluN1, and the GluN2 subunit that the GluN1 joins with.^8,9^ GluN2 subunits are found to contribute to different physiological and pharmacological properties of the NMDA receptor. GluN2A (or called NR2A) and GluN2B play an important role in synaptic plasticity, not only in cortices but throughout the brain. In forebrain structures including the ACC, GluN2A and GluN2B subunits are two major NMDA GluN2 isoforms. GluN2A and GluN2B subunits show distinct properties to NMDA receptors; receptors containing GluN1 plus GluN2B mediate a current that decays three to four times more slowly than receptors composed of GluN1 plus GluN2A.^3^

## Pharmacological and genetic investigation of GluN2B receptors in postsynaptic long-term potentiation

**Figure 1.**
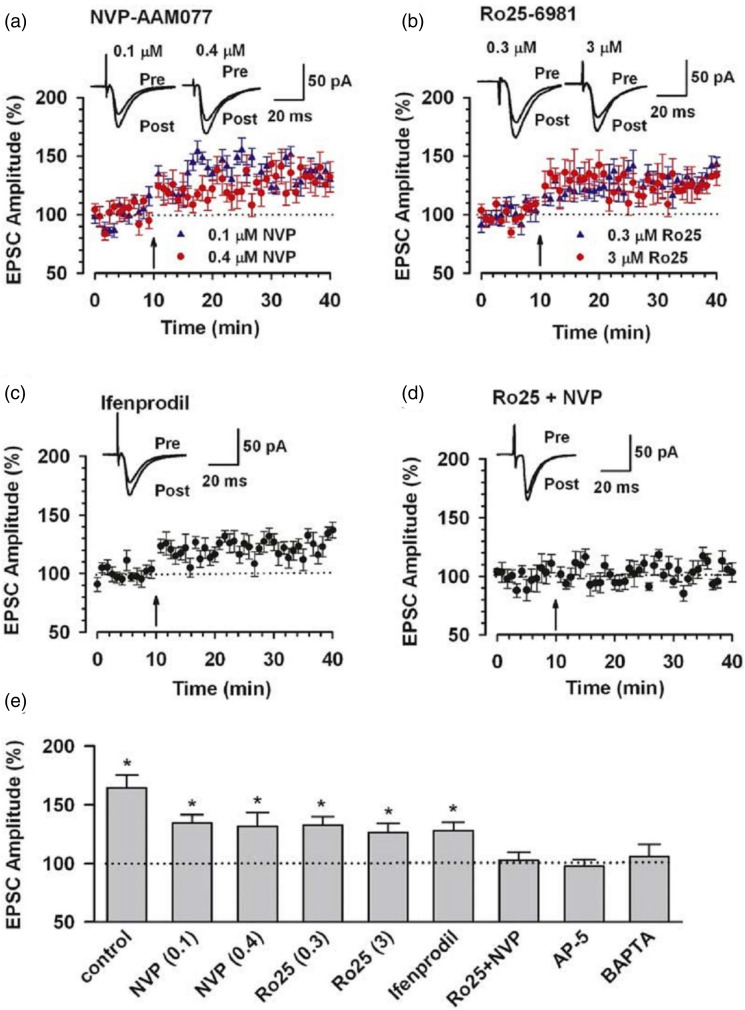
Contributions of the GluN2A and GluN2B Subunits to the induction of LTP in the ACC (a) LTP induced by the pairing training was partially depressed by 0.1 μM (*n* = 7) or 0.4 μM (*n* = 9) NVP-AAM077. (b) LTP was partially depressed by 0.3 μM (*n* = 9) or 3 μM (*n* = 5) Ro25-6981. (c) LTP was partially depressed by 3 μM ifenprodil (*n* = 7). (d) The coapplication of 0.4 μM NVP-AAM077 and 0.3 μM Ro25-6981 completely blocked LTP (*n* = 6). (a)–(d) The insets show averages of six EPSCs 5 min before and 25 min after the pairing training (arrow). The dashed line indicates the basal synaptic responses. (e) Summary of the effects of NMDA receptor subunit antagonists or postsynaptic injection of BAPTA on LTP. *p < .05 compared to baseline. (Adapted from Zhao et al.,^15^).

One typical method to investigate the roles of GluN2B is by using selective receptor antagonists. By inhibiting GluN2B mediated NMDA receptors, it has been reported that LTP induction at various central synapses requires the activation of GluN2B-NMDA receptors.^3^ Because the NMDA receptor-mediated currents are mainly carried out by GluN2A-containing NMDA receptors, inhibition of the GluN2B receptor alone may not be sufficient to produce complete blockade of LTP. In the ACC excitatory synapses, LTP induced by the pairing protocol (paired presynaptic 80 pulses at 2 Hz with postsynaptic depolarization at +30 mV)^10^ was significantly reduced but not blocked by GluN2B antagonists. Interestingly, contrast to the pairing protocol, Zhao et al reported that LTP in the ACC induced by spike-timing (paired three presynaptic stimuli that caused three EPSPs (10 ms ahead) with three postsynaptic APs at 30 Hz, paired 15 times every 5 s) is completely blocked by GluN2B antagonist (Figure 1). It is still unclear in what way different induction protocols may mimic different physiological/pathological functions. The expression of this NMDA receptor-dependent LTP is due to postsynaptic AMPA receptors.^11,12^

Due to lack of a selective GluN2B enhancer, there is no report of the effect of enhancing GluN2B functions. One way to test this is to create transgenic mice with forebrain overexpression of GluN2B subunits. In a previous study using genetic overexpression of GluN2B subunits, it was found that LTP induced by weak tetanic stimulation was significantly enhanced in the CA1 region of the hippocampus.^13^ In the ACC, NMDA receptor responses are enhanced, and activation of plasticity-related immediate early genes was significantly enhanced by peripheral inflammation.^1^ These findings provide consistent evidence for the contribution of GluN2B to LTP and its related cortical excitation.

## NMDA receptor-independent presynaptic long-term potentiation

There are at least two forms of LTP in ACC synapses: postsynaptic LTP (post-LTP, an NMDA receptor-dependent LTP) and presynaptic LTP (pre-LTP, an NMDA receptor-independent LTP). While the NMDA receptor contributes to the induction of post-LTP, pre-LTP does not require activation of the NMDA receptor at all. With the presence of a NMDA receptor antagonist AP-5, low-frequency stimulation (2 Hz) induces the pre-LTP in the ACC.^14^ The activation of presynaptic glutamate kainate (KA) receptor is critical for pre-LTP. In ACC slices obtained from mice lacking the KA GluK1 subunit, this pre-LTP is completely blocked. The requirement for GluK1 is selective, pre-LTP is not affected in ACC slices of mice lacking the GluK2 subunit. The requirement of GluK1 for pre-LTP in the ACC is further confirmed by pharmacological results, since a potent GluK1-selective KA receptor antagonist UBP310 blocked the induction of pre-LTP. In addition to GluK1 receptor, the L-type voltage-gated calcium channels (L-VGCCs) have been found to contribute to the induction of pre-LTP. A selective antagonist, nimodipine, reduced the amplitude of pre-LTP in the ACC. Intracellularly, the pre-LTP in the ACC requires activation of the cAMP signalling pathway, including calcium-stimulated adenylyl cyclase subtype 1 (AC1).^15^ It is likely that cAMP-related signaling pathways enhance the release of glutamate during pre-LTP. The maintenance or expression of ACC pre-LTP requires the modulation of hyperpolarization-activated cyclic nucleotide-gated (HCN) channels.^14,16^ Future studies are needed to determine molecular mechanism for the enhancement of glutamate release.

## NMDA receptors and long-term depression

Recent studies have characterized two major forms of LTD in the ACC based on their sensitivity to different pharmacological inhibitors. One form of LTD requires the activation of mGluR1 and L-VGCCs.^1^ Glutamate NMDA receptors do not significantly affect the induction of this form of LTD.^17^ The other form of LTD is induced by pairing protocols. This form of LTD requires activation of NMDA receptors. Pharmacological experiments using different GluN2 subunit antagonists found that both GluN2A- and GluN2B receptors contribute to this LTD. Neither GluN2A nor GluN2B is absolutely required, as LTD can be rescued by altering holding potentials at postsynaptic neurons.^18^ This form of LTD also requires a postsynaptic increase in postsynaptic Ca^2+^ and CaM levels.^18,19^ NMDA receptor-dependent LTD is mediated by postsynaptic changes of AMPA receptors, and the signaling protein(s) interacting with the C-terminal tail of the GluA2 subunit are important. This is supported by a genetic study which demonstrated that ACC LTD is abolished in mice lacking GluA2 but is intact in mice lacking GluA3. NMDA receptor-dependent LTD is also blocked by another exogenous peptide that interferes with the interaction between GluA2 and N-ethylmaleimide-sensitive fusion protein (NSF), suggesting that displacement of NSF by the clathrin–adaptor protein two complex is also involved in NMDA receptor-dependent LTD in the ACC.^19^ Less research has been done on NMDA receptor-independent LTD, since it is more readily obtained with field recordings. A recent study in the ACC further investigated synaptic depotentiation after the induction of LTP and found that the activation of NMDA GluN2B containing receptor is selectively required for synaptic depotentiation. By contrast, GluN2A containing receptor is not required.^20^

## GluN2B and chronic pain

**Figure 2.**
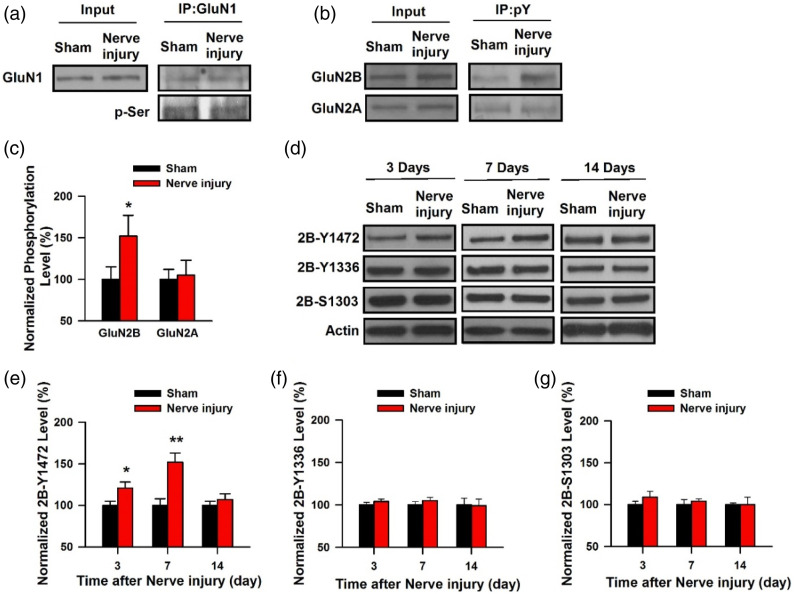
GluN2B-Y1472 is involved in the upregulation of NMDA receptor in the IC (a) The serine phosphorylation level of the GluN1subunit showed no change 7 days after nerve injury surgery (*n* = 4, *p* > .05). (b) and (c) The tyrosine phosphorylation of the GluN2B subunit, but not the GluN2A subunit, was increased on day 7 after nerve injury (*n* = 4 for each group). (d) Western blots for 2B-Y1472, 2B-Y1336, and 2B-S1303 in the PSD-enriched fraction of the IC obtained 3 days and 2 weeks after nerve injury or the sham control group. (e) The level of 2B-Y1472 was significantly increased on day 3 and day 7, but not on day 14, after nerve injury compared with the sham control (*n* = 6 for each group). (f) The level of 2B-Y1336 showed no change on days 3, 7 or 14 after nerve injury compared with the sham control (*n* = 6 for each group). (g) The level of 2B-S1303 showed no change on day 3, 7 or 14 after nerve injury compared with sham control (*n* = 6 for each group) *indicates *p* < .05; **indicates *p* < .01 compared with sham control; error bars indicate SEMs. (Adapted from Qiu et al.,^24^).

In different animal models of chronic pain, it has been reported that glutamate mediated excitatory synaptic transmission in the ACC was significantly increased after the injury.^5,21^ Presynaptically, the release of glutamate is significantly enhanced. At the postsynaptic site, AMPA receptor-mediated responses are increased. Biochemically, this is in part due to the phosphorylation of AMPA receptors by cAMP-dependent protein kinase (PKA).^22^ Differing from synaptic LTP in the spinal cord, NMDA receptors including GluN2B are also increased in the forebrains. The increased expression of NMDA GluN2B receptors and GluN2B receptor-mediated synaptic currents were detected in the pyramidal cells of ACC of adult mice.^23^ In IC, peripheral nerve injury caused a long-lasting increase in the amount of synaptic NMDA receptors without any significant changes in extrasynaptic NMDA receptors. The phosphorylation of the NMDA receptor subunit GluN2B at Tyr (1472) by a pathway involving AC1, PKA, and Src family kinases is required for the upregulation of NMDA GluN2B receptor (see Figure 2).^24^ Microinjection of a selective GluN2B-specific antagonists into the insular cortex produced significant inhibition of behavioral allodynic responses to non-noxious stimuli in the mouse model of neuropathic pain.^24^ This new finding is consistent with previous genetic studies with GluN2B overexpression in the forebrains.^25^

In a genetic study using forebrain selective overexpression of NMDA GluN2B subunit, it was demonstrated that behavioral sensitization triggered by peripheral inflammation of formalin and complete Freund’s adjuvant were significantly enhanced in transgenic mice.^25^ These results provide the first genetic evidence that forebrain NMDA GluN2B contributes to chronic pain. Subsequent pharmacological studies using NMDA GluN2B selective antagonists further confirmed that inhibition of ACC NMDA receptor GluN2B produced significant analgesic effects in animal models of chronic pain.^3,26^

## GluN2B and its downstream adenylyl cyclase subtype 1 in cortical synapses

**Figure 3.**
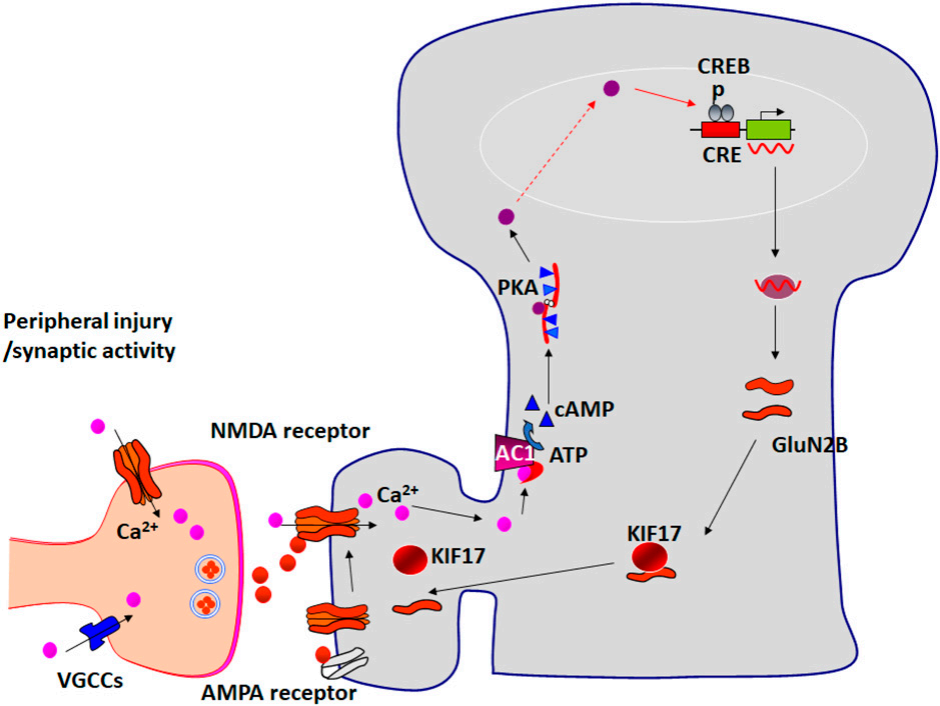
Positive feedback control of GluN2B in cortical synapses. Glutamate is the major fast excitatory transmitter in the ACC and IC. Peripheral injury (e.g., tissue inflammation or nerve injury) triggers a burst of abnormal activity in the cortical circuits. Activation of NMDA receptor induces calcium (Ca^2+^) influx into the postsynaptic site. Postsynaptic increases in Ca^2+^ lead to activation of Ca^2+^-calmodulin (CaM) dependent pathways. Among them, Ca^2+^ and CaM stimulated AC1 is activated, and this activation leads to the generation of the second messenger cAMP. Subsequently, cAMP activates PKA. PKA subunit then translocates to the nucleus and phosphorylates CREB. GluN2B contains a CREB binding domain which may couple increases in intracellular Ca^2+^ with the increase in GluN2B expression. Consequently, postsynaptic synthesis of NMDA GluN2B is increased. With the help of endogenous motor protein KIF17, these new NR2B subunits are moved to synaptic space and added to postsynaptic NMDA receptors. In addition to its postsynaptic roles, NMDA receptors are also existed at presynaptic terminals, and may modulate the release of glutamate in the cortex. The NMDA receptor may bring outside Ca^2+^ to presynaptic terminals, in addition to those induced by voltage-gated calcium channels (VGCCs).

Cumulative studies have consistently indicated that calcium-stimulated AC1 acts as a downstream signalling protein in NMDA receptor dependent synaptic plasticity, as well as in injury-induced changes. For example, AC1 is important for NMDA receptor-triggered postsynaptic AMPA receptor upregulation in the ACC. Furthermore, in vivo biochemical studies reveal that AC1 activity is required for NMDA receptor GluN2B upregulation caused by peripheral visceral pain information.^27^ Since GluN2B-containing NMDA receptors are important for AC1 activation, this singling pathway forms a positive feedback loop on cortical synapses. Activation of GluN2B-containing NMDA receptor led to the activation of AC1, and the production of cAMP. Activation of PKA and CREB triggers the new synthesis of GluN2B. The increased GluN2B enhanced NMDA receptor mediated responses, and positively increases cAMP. This positive feedback loop serves to maintain cortical excitation in pathological pain conditions.^27^ Figure 3 summaries the signaling pathway for the upregulation on cortical synapses.

## Presynaptic NMDA receptors

Aside from the postsynaptic NMDA receptors, presynaptic NMDA receptors have also been reported to modulate glutamate release, and extra-synaptic NMDA receptors are involved in presynaptic regulation.^28–31^ Presynaptic NMDA receptors may regulate neurotransmitter release by (1) directly, or indirectly, causing Ca^2+^ influx to trigger vesicle exocytosis, or (2) modulating downstream intracellular signaling cascades through metabotropic effects that are independent of Ca^2+^ flux. Presynaptic NMDA receptors can also be involved in postsynaptically expressed LTP/LTD, as described in the cerebellum at synapses between parallel fiber and Purkinje cells.^32^ In a recent study, Chen et al. reported that the GluN2C/2D modulates the presynaptic glutamate release in the ACC.^30^ The role of presynaptic NMDA receptors in spontaneous release also requires activation of the c-JunN-terminal kinase 2 (JNK2) pathway.^33^ The presynaptic NMDA receptors in spontaneous and evoked release work in a nonoverlapping manner to modulate the presynaptic release. Future studies are needed to understand potential contribution of NMDA receptors to other forms of regulation and plasticity.

## Conclusion and future directions

In conclusion, it is clear that GluN2B containing NMDA receptors play an important role in central plasticity. Genetic and pharmacological approaches provide powerful tools to understand their function in the central synapses and physiological functions. More importantly, it may also serve as a potential drug target to prevent or help patients with memory loss or chronic pain. We will need more work to develop new medicine that can treat diseases without the loss of major physiological functions such as memory and cognition.
